# The Pro-Inflammatory Action of Different Pathogen-Associated Molecular Patterns in Porcine Hepatic and Intestinal Cell Cultures

**DOI:** 10.3390/ani16121777

**Published:** 2026-06-09

**Authors:** Gábor Mátis, Andrea Lajos, Rege Anna Márton, Ágnes Kemény, Zsuzsanna Neogrády, Máté Mackei

**Affiliations:** Division of Biochemistry, Department of Physiology and Biochemistry, University of Veterinary Medicine, István u. 2, H-1078 Budapest, Hungary; lajos.andrea@univet.hu (A.L.); marton.rege.anna@univet.hu (R.A.M.); kemeny.agnes@univet.hu (Á.K.); neogrady.zsuzsanna@univet.hu (Z.N.); mackei.mate@univet.hu (M.M.)

**Keywords:** swine, cell cultures, infectious diseases, inflammatory cytokines, oxidative stress, antibiotic alternatives

## Abstract

The reduction of antibiotic use in livestock production necessitates the development of reliable in vitro models for evaluating alternative immunomodulatory compounds. In this study, porcine hepatocyte–non-parenchymal (NP) cell co-cultures and small intestinal explants exposed to pathogen-associated molecular patterns (PAMPs), including lipopolysaccharide (LPS), lipoteichoic acid (LTA), flagellin and polyinosinic–polycytidylic acid (poly I:C), were established as inflammatory models. Hepatic co-cultures exhibited pronounced inflammatory responses, with LPS and LTA increasing hepatocellular interleukin (IL)-4, IL-6, IL-8 and tumor necrosis factor (TNF)-α release, alongside elevated extracellular lactate dehydrogenase activity and maintained metabolic activity, indicating membrane perturbation without marked cytotoxicity. Poly I:C was found to be cytotoxic, accompanied by an altered cytokine profile. All PAMPs enhanced reactive oxygen species production without remarkable lipid peroxidation in most cases. In contrast, intestinal explants showed high resilience with unchanged viability and limited cytokine responses. Only IL-6 and IL-8 were detectable, with LPS elevating IL-8, while poly I:C and flagellin increased IL-6 levels. These findings demonstrate marked tissue-specific differences in inflammatory responsiveness. The hepatic co-culture model provides a sensitive system for studying robust inflammatory reactions, whereas intestinal explants are suitable for investigating moderate, gut-specific immune responses. In conclusion, these complementary models offer valuable tools for evaluating antibiotic alternatives in swine.

## Simple Summary

The overuse of antibiotics in pig farming is a growing concern because it contributes to drug-resistant pathogens, making it harder to treat infectious diseases in both animals and humans. To reduce antibiotic use, new alternatives need to be developed and tested. This study aimed to establish laboratory models using porcine liver cells and small intestinal tissue to mimic the inflammatory response triggered by bacterial and viral infections. These models were exposed to compounds commonly present in pathogenic bacteria and viruses to evoke inflammation and oxidative stress. It was found that liver cells reacted strongly, releasing high levels of inflammation-associated molecules without major cellular damage, while intestinal tissue showed a milder and more controlled response. These results highlight important differences between the responses of liver and intestinal tissues to inflammatory stimuli. The study concludes that the two developed models can be well applied to test novel and safer alternatives to antibiotics. This approach can help reduce antibiotic use in livestock, improve animal health and contribute to limiting the spread of antibiotic resistance, which is a major global health challenge.

## 1. Introduction

In pigs raised under intensive conditions, the risk of infections—particularly of gastrointestinal and respiratory origin—is markedly increased. Rapid weight gain, crowding, enteric infections and transport-related or heat stress can all contribute to health concerns, including gut microbiome dysbiosis, which in turn may result in immune suppression, reduced production indices and elevated mortality rates [1].

Studying pathogen-associated inflammatory responses is essential for developing novel immunomodulatory strategies in swine to combat the emerging antibiotic resistance. Such investigations require the use of cell or tissue culture models, in which inflammatory responses can be induced in vitro to a degree comparable to systemic or enteric infections in vivo. Inflammatory stimulation of hepatocyte-derived cell cultures provides a reliable means of modeling the liver’s central role as an immune organ in systemic inflammation [2]. Mimicking intestinal inflammation is more challenging due to the complex functions and histological structure of the gut. In vitro systems composed solely of epithelial cells cannot fully reproduce inflammatory processes since intestinal barrier integrity relies on more than epithelial cell-to-cell junctions [3]. By establishing ex vivo explant cultures that more accurately reflect the complex histological organization of the small intestine, it becomes possible to obtain a more comprehensive insight into the intestinal mucosal immune response and to evaluate redox homeostasis, an important factor associated with inflammation [4].

Based on the previous work of our research group, significant results were achieved in modeling inflammation through the establishment of primary hepatic cell cultures and ex vivo explant cultures from chickens [5,6]. These findings indicate that primary cell and tissue culture systems can serve as useful platforms for characterizing host inflammatory and redox responses under controlled experimental conditions. Some porcine hepatic in vitro models, such as primary two- or three-dimensional hepatocyte cultures and hepatocyte–Kupffer cell co-cultures [7,8,9] or the PICM-19 embryonic liver cell line [10], have already been developed; however, further complementary inflammatory model systems are still needed to characterize the PAMP-induced hepatic responses. Concerning porcine small intestinal models, the non-tumorigenic jejunal IPEC-J2 cell line is widely applied [11], and organoid-based models have also been introduced [12,13]. However, there is still a need for improved tissue-based systems suitable for monitoring infection-associated gut inflammation. Although immortalized intestinal epithelial cell lines such as IPEC-J2 are reproducible and easy to maintain, explant cultures better preserve the native multicellular architecture and local microenvironment of the intestinal mucosa, despite their shorter sustainability and increased biological variability [3,12].

Inflammatory responses can be effectively triggered in vitro by applying pathogen-associated molecular patterns (PAMPs). These molecules, including bacterial cell wall components, flagellar proteins and synthetic nucleic acid analogs mimicking viral replication intermediates, interact with cell surface receptors, mostly toll-like receptors (TLRs), and activate inflammatory signaling pathways. Lipopolysaccharides (LPS), as endotoxins of Gram-negative bacteria, are recognized by TLR4, while lipoteichoic acid (LTA), considered a Gram-positive endotoxin, activates the TLR2 pathway, enterobacteria-originated flagellin stimulates TLR5, whereas the viral double-stranded RNA analogue polyinosinic–polycytidylic acid (poly I:C) is detected by TLR3 [13,14,15]. These interactions lead to the activation of downstream signaling cascades and the subsequent production of pro-inflammatory cytokines, including interleukin (IL)-2, IL-6, IL-8, tumor necrosis factor (TNF)-α and type I interferons, depending on the exact TLR pathway [16]. These cytokines recruit and activate immune cells, influence one another and enable communication between immune and non-immune cells, thereby coordinating the immune response [17]. Inflammatory processes are largely coupled to oxidative stress due to the depletion of antioxidant systems, the accumulation of reactive oxygen species (ROS) and the disruption of cellular redox homeostasis [18]. The hepatic and intestinal inflammatory response coupled to different PAMPs has already been studied in chicken cell culture models by our research group [2,6]; however, species-specific differences in TLR signaling, in particular between mammals and birds [19,20], underline the importance of further investigations in swine.

In the present study, the aim was to establish reliable in vitro inflammatory models of porcine origin, applying primary hepatocyte–non-parenchymal (NP) cell co-cultures and small intestinal explants. Our major goal was to monitor viability and assess the cytokine response triggered by various PAMPs in these cell and tissue cultures, which may serve as model systems for studying the hepatic and enteral inflammatory response in swine, while also allowing comparison with analogous chicken-derived primary models previously established by our research group.

## 2. Materials and Methods

### 2.1. Isolation and Culturing of Hepatic Cells

All reagents and chemicals utilized throughout the study were acquired from Merck (Darmstadt, Germany) unless otherwise specified. Hepatic cells were isolated based on Mátis et al. 2017 [8] from a post-weaning castrated male pig (Hungarian Large White breed, 15 kg body weight), originating from Dunahyb Ltd. (Szekszárd, Hungary) and slaughtered under standard conditions in a commercial slaughterhouse (Keselyűs-Hús Kft., Szekszárd, Hungary). The caudate lobe of the liver was flushed with a three-step ex vivo perfusion with buffer solutions injected directly into the lobe via the large intrahepatic vessels on the cut surface. All buffers were freshly oxygenated with Carbogen (95% O_2_, 5% CO_2_); the flow rate was set to 100 mL/min. In the first step, 400 mL of chilled Hanks’ Balanced Salt Solution (HBSS) containing ethylenediamine–tetraacetic acid (EDTA, 1 g/L) was used, followed by 500 mL of EDTA-free HBSS (pre-warmed to 37 °C), and finally, the liver structure was digested with 300 mL of HBSS supplemented with type IV collagenase (1 g/L [Nordmark, Uetersen, Germany]; containing 7 mM CaCl_2_ and 7 mM MgCl_2_, pre-warmed to 37 °C). Once the Glisson’s capsule was excised, the digested liver parenchyma was filtered through three layers of sterile gauze with a pore size of 100 µm to remove cell aggregates. The resulting primary cell suspension was incubated in HBSS buffer containing 2.5% bovine serum albumin (BSA) for 75 min, thereafter, it was centrifuged three times (100× *g*, 75 s, 4 °C), resuspending the sediment in Williams’ Medium E (supplemented with 15 mM of 4-[2-hydroxyethyl]-1-piperazineethanesulfonic acid [HEPES], 1% penicillin-streptomycin, 2 mM glutamine, 20 IU/L insulin, 0.5 µmol/mL amphotericin-B and 10% fetal bovine serum [FBS, heat inactivated]) to gain the hepatocyte-enriched fraction.

The supernatant, containing NP cells, was centrifuged at 500× *g* for 5 min. The resulting pellet was resuspended in 10 mL of supplemented Williams’ Medium E and layered onto two layers of Percoll (15 mL of 50% Percoll and 20 mL of 25% Percoll). Following centrifugation at 500× *g* for 20 min, the NP cell fraction—enriched primarily in macrophages, particularly Kupffer cells—was recovered from the interface between the two Percoll layers. This cell suspension was aspirated and centrifuged at 600× *g* for 20 min, and the final pellet was resuspended in 5 mL of supplemented Williams’ Medium E. This isolation procedure was based on our previously established and standardized porcine hepatic co-culture model, in which the macrophage enrichment of the NP fraction and the cell ratio of confluent co-cultures were confirmed by immunohistochemical detection of the macrophage-specific CD68 antigen [8].

The trypan blue exclusion test was used to determine cell count and viability. The cell concentrations of both fractions were adjusted to 4 × 10^5^/mL after cell counting using a Bürker chamber (Brand GmbH, Wertheim, Germany). Hepatocyte and NP cell fractions were mixed in a ratio of 6:1. The cell suspension was seeded into 24- and 96-well culture dishes (Greiner Bio-One, Frickenhausen, Germany) (300 µL and 100 µL per well, respectively) pre-coated with type I collagen and incubated for 4 h at 37 °C with 5% CO_2_. The culture medium was changed 4 h following cell seeding to fresh, supplemented Williams’ Medium E. After 24 h, single-layer confluent cultures were formed, being ready for subsequent treatments.

### 2.2. Isolation and Culturing of Intestinal Explants

To obtain intestinal explants, a 15 cm long segment of the ileum, starting 10 cm proximally to the ileocecal junction, was excised and immediately placed into ice-cold phosphate-buffered saline (PBS) containing 1% penicillin-streptomycin, used as a washing solution. Following rinsing of the external surface, the intestinal content was removed, and the gut section was washed three times. After longitudinal incision along the mesenteric border, the mucosal surface was rinsed, and the intestinal tissue was stretched onto a sterile glass plate cooled on ice using a microscope slide, while being kept continuously moist with the washing solution. Explant samples with a diameter of 1.5 mm were excised from the mucosa using a biopsy punch (MDE GmbH; Heidelberg, Germany). The explants were placed into the wells of a 96-well culture plate (Greiner Bio-One, Mosonmagyaróvár, Hungary) coated with collagen type I, each well containing 200 µL of Dulbecco’s Modified Eagle’s Medium/Nutrient F-12 Ham (DMEM–F12) culture medium supplemented with 2.5% FBS, 1% glutamine; 1% penicillin-streptomycin and a growth factor mixture supplement composed of a single dose of HCMTM SingleQuotsTM Kit (Lonza-Biocenter, Szeged, Hungary). After 2 h of incubation (37 °C, 5% CO_2_), intestinal explant cultures were ready for treatments.

### 2.3. Treatments of Cell Cultures

Treatments were performed for 24 h in the case of hepatic cell cultures and for 12 h in the case of intestinal explants. These exposure times were selected according to the different biological characteristics and maintainability of the two model systems. Hepatic co-cultures formed stable confluent monolayers and allowed a 24 h treatment period, whereas intestinal explants showed limited long-term sustainability; therefore, a shorter 12 h exposure was applied to preserve tissue viability and histological structure, similar to our previous studies with chicken-derived cell and tissue cultures [2,6].

The culture media were supplemented with 10 or 50 µg/mL LPS from *Escherichia coli* (O55:B5), 10 or 50 µg/mL of LTA from *Staphylococcus aureus*, 100 or 250 ng/mL flagellin from *Salmonella* Typhimurium, and 50 or 100 µg/mL poly I:C (*n* = 6 per group in all cases). Poly I:C was heated to 50 °C for 3 min to facilitate proper dissolution and then cooled down for re-annealing, ensuring the preparation of a reproducible double-stranded RNA analogue before being added to the cell culture medium. The applied concentrations were selected based on previous studies using comparable porcine and chicken hepatic or intestinal in vitro models [2,6,21].

After the exposure period, samples were taken from the culture media of hepatic co-cultures in 24-well dishes, and cells were thereafter lysed by applying 50 µL/well Mammalian Protein Extraction Reagent (M-PER). Metabolic activity of cultured cells was assessed by the Cell Counting Kit (CCK)-8 assay in 96-well plates. Regarding the intestinal explants, culture medium samples were first obtained from 96-well dishes, and the CCK-8 test was performed thereafter. All supernatant and cell lysate samples were stored at −80 °C until laboratory measurements.

### 2.4. Laboratory Measurements

The metabolic activity of hepatic cell cultures and intestinal explants was monitored using the CCK-8 assay (Dojindo Molecular Technologies, Rockville, MD, USA), according to the manufacturer’s instructions. Following the applied treatments, 100 µL fresh medium supplemented with 10 µL CCK-8 reagent was added to each well, and the cells were incubated for 2 h at 37 °C under the conditions described previously. After incubation, the medium was transferred to a new 96-well plate, and the absorbance was measured at 450 nm using a Multiskan GO 3.2 reader (Thermo Fisher Scientific, Waltham, MA, USA).

Extracellular lactate dehydrogenase (LDH) activity, indicating membrane integrity, was determined from culture media, using a kinetic colorimetric LDH assay kit (Diagnosticum Ltd., Budapest, Hungary). Following the manufacturer’s instructions, 8 µL (hepatic cultures) and 25 µL (intestinal cultures) media were adjusted to a final volume of 50 µL with the appropriate dilution buffer, followed by the addition of 50 μL nicotinamide adenine dinucleotide (NAD^+^)-containing freshly prepared Master Reaction Mix. The assay was carried out at 37 °C; absorbance values were first measured 2 min after starting the assay, followed by further measurements in 5 min intervals at 450 nm with a Multiskan GO 3.2 reader (Thermo Fisher Scientific Inc., Waltham, MA, USA) until the value of the most concentrated standard was exceeded by the most active sample. Enzyme activity was calculated using the equation provided by the manufacturer.

For liver cell cultures, the fluorometric Amplex Red technique (Thermo Fisher Scientific, Waltham, MA, USA; Cat. Nr.: A22188) was used to measure H_2_O_2_ levels in the culture medium. 50 µL of freshly prepared Amplex Red Working Solution was mixed with 50 µL of culture medium, incubated at room temperature (24 °C) for 30 min in the absence of light; fluorescence (ex = 530 nm, em = 590 nm) was detected using a Victor X2 2030 fluorometer (Perkin Elmer, Waltham, MA, USA). The concentration of malondialdehyde (MDA) as a marker of lipid peroxidation was measured according to the manufacturer’s instructions with a specific colorimetric assay (Cat. Nr.: MAK085). 300 µL of freshly prepared thiobarbituric acid stock solution was mixed with 100 µL of cell lysate according to the manufacturer’s protocol, and the solution was incubated at 95 °C for 1 h and then cooled on ice for 10 min. Then the absorbance was measured at 532 nm using a Multiskan GO 3.2 reader.

The concentrations of IL-4, IL-6, IL-8, TNF-α, granulocyte–monocyte colony-stimulating factor (GM-CSF) and interferon (IFN)-γ were measured by applying the Luminex xMAP method, using the Milliplex Porcine Cytokine/Chemokine Panel (Cat. Nr.: PCYTMAG-23K) following the manufacturer’s instructions. A 96-well plate was loaded with 25 μL aliquots of culture medium samples in duplicates. Subsequently, 25 μL of six distinct sets of colored, capture antibody-conjugated beads were added to each well, followed by an overnight incubation period and subsequent washing steps. In the next phase, biotinylated detection antibodies and streptavidin-phycoerythrin solutions were applied. The plate was then supplemented with 150 μL of drive fluid, and the beads were resuspended by agitation on a plate shaker for 5 min. Finally, the measurement was performed using a Luminex MAGPIX instrument, and the resulting data were processed with the Luminex xPonent 4.2 software. Standard curves for all analytes were generated using the Belysa Immunoassay Curve Fitting software 1.2.2 based on the median fluorescence intensity values. All reported cytokine concentrations were within the linear detection range of the assay.

### 2.5. Statistics

Statistical analyses were performed using the R 4.5.3 software (R Core Team, 2020). Data showed normal distribution as confirmed using the Shapiro–Wilk test and homogeneity of variances was verified using Levene’s test for each parameter; hence, one-way analysis of variance (ANOVA) and Dunnett’s post-hoc tests were used for statistical evaluation. In all cases, the values of the treated groups were compared to those of the controls. If the *p*-value was less than 0.05, the difference was considered significant. Graphs were generated using the GraphPad Prism 9 software (GraphPad Software Inc., San Diego, CA, USA).

## 3. Results

### 3.1. Hepatic Cell Cultures

**Figure 1:**
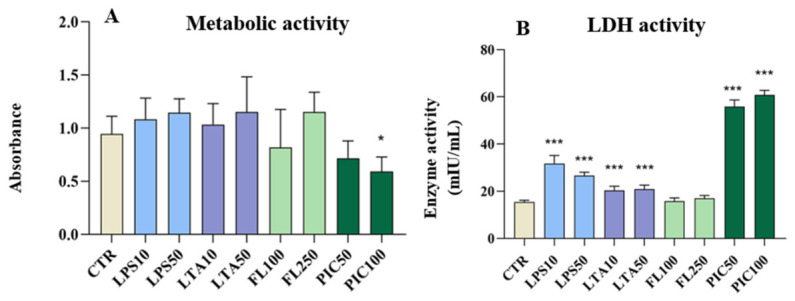
(**A**) Metabolic activity and (**B**) extracellular lactate dehydrogenase (LDH) activity of porcine hepatocyte–non-parenchymal cell co-cultures, assessed with the CCK-8 test (**A**) and a kinetic photometric assay (**B**). Absorbance (**A**) and enzyme activity (mIU/mL, (**B**) values are indicated on the y axes as mean + standard error of the mean. LPS10 and LPS50 refer to 10 and 50 µg/mL lipopolysaccharide (LPS) from *Escherichia coli* (O55:B5), LTA10 and LTA50 indicate 10 and 50 µg/mL lipoteichoic acid (LTA) from *Staphylococcus aureus*, FL100 and FL250 denote 100 or 250 ng/mL flagellin from *Salmonella* Typhimurium, while PIC50 and PIC100 indicate 50 and 100 µg/mL polyinosinic–polycytidylic acid (poly I:C) exposures. *n* = 6 per group in all cases. * *p* < 0.05 and *** *p* < 0.001 compared to the controls.

The applied treatments did not significantly affect cellular metabolic activity, except for the higher dose of poly I:C, which resulted in a significant reduction (*p* = 0.033) (Figure 1A). The extracellular LDH activity measured in the culture medium showed a significant increase following exposure to both concentrations of LPS, LTA and poly I:C (*p* < 0.001 in all cases) (Figure 1B).

**Figure 2:**
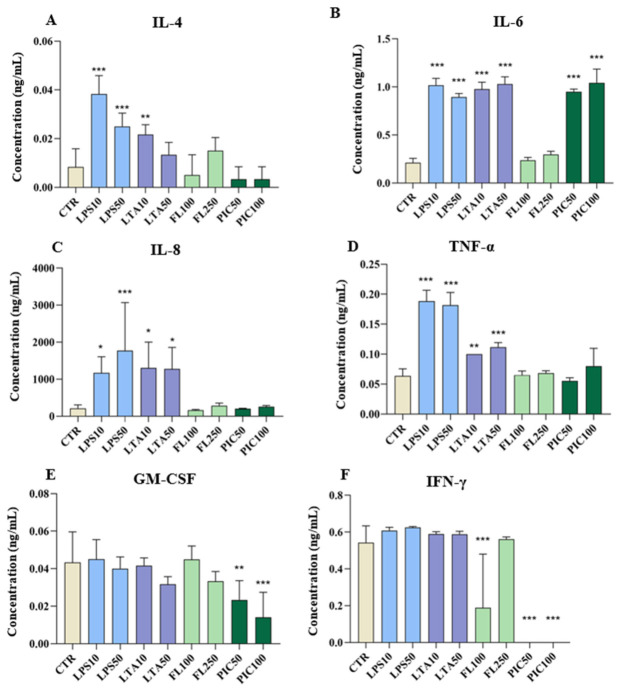
The extracellular concentrations of (**A**) interleukin (IL)-4, (**B**) IL-6, (**C**) IL-8, (**D**) tumor necrosis factor (TNF)-α, (**E**) granulocyte–monocyte colony-stimulating factor (GM-CSF) and (**F**) interferon (IFN)-γ of porcine hepatocyte–non-parenchymal cell co-cultures, assessed with Luminex xMAP technology. Concentrations (ng/mL) are indicated on the y-axes as mean + standard error of the mean. LPS10 and LPS50 refer to 10 and 50 µg/mL lipopolysaccharide (LPS) from *Escherichia coli* (O55:B5), LTA10 and LTA50 indicate 10 and 50 µg/mL lipoteichoic acid (LTA) from *Staphylococcus aureus*, FL100 and FL250 denote 100 or 250 ng/mL flagellin from *Salmonella* Typhimurium, while PIC50 and PIC100 indicate 50 and 100 µg/mL polyinosinic–polycytidylic acid (poly I:C) exposures. *n* = 6 per group in all cases. * *p* < 0.05, ** *p* < 0.01 and *** *p* < 0.001 compared to the controls.

Among the inflammatory cytokines, extracellular IL-4 concentrations of hepatic co-cultures were significantly increased following treatment with both doses of LPS (*p* < 0.001 in both cases) and after being exposed to 10 µg/mL LTA (*p* = 0.003) (Figure 2A). The IL-6 concentrations of culture media showed a significant elevation in response to all applied doses of LPS, LTA and poly I:C (*p* < 0.001 in each case) (Figure 2B). Furthermore, IL-8 secretion of cultured cells incubated with both concentrations of LPS (*p* = 0.038 and *p* < 0.001) and LTA (*p* = 0.014 and *p* = 0.017) was significantly enhanced compared to the untreated control group (Figure 2C). Exposure to LPS and LTA at both 10 and 50 µg/mL concentrations likewise resulted in elevated TNF-α levels in the supernatant (*p* < 0.001 for both LPS doses; *p* = 0.001 and *p* < 0.001 in the case of LTA10 and LTA50 groups, respectively) (Figure 2D). The extracellular concentration of GM-CSF was significantly reduced by both lower (*p* = 0.006) and higher (*p* < 0.001) doses of poly I:C (Figure 2E), while IFN-γ secretion of hepatic cells was significantly decreased following treatments with 250 ng/mL flagellin and both concentrations of poly I:C (*p* < 0.001 in all three cases) (Figure 2F).

**Figure 3:**
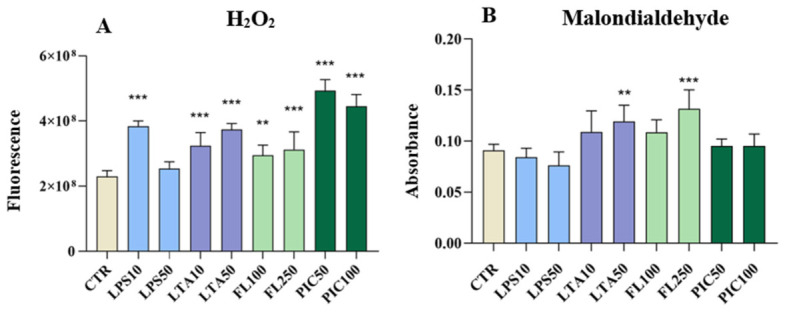
(**A**) The extracellular concentration of H_2_O_2_ and (**B**) the intracellular concentration of malondialdehyde (MDA) of porcine hepatocyte–non-parenchymal cell co-cultures, assessed with the fluorometric Amplex Red technique (**A**) and a specific colorimetric assay (**B**). Fluorescence (**A**) and absorbance (**B**) values are indicated on the y-axes as mean + standard error of the mean. LPS10 and LPS50 refer to 10 and 50 µg/mL lipopolysaccharide (LPS) from *Escherichia coli* (O55:B5), LTA10 and LTA50 indicate 10 and 50 µg/mL lipoteichoic acid (LTA) from *Staphylococcus aureus*, FL100 and FL250 denote 100 or 250 ng/mL flagellin from *Salmonella* Typhimurium, while PIC50 and PIC100 indicate 50 and 100 µg/mL polyinosinic–polycytidylic acid (poly I:C) exposures. *n* = 6 per group in all cases. ** *p* < 0.01 and *** *p* < 0.001 compared to the controls.

Concerning the redox parameters assessed, extracellular H_2_O_2_ levels were significantly elevated following treatment with 10 µg/mL LPS, as well as after exposure to all tested doses of LTA, flagellin and poly I:C (*p* = 0.008 for 100 ng/mL flagellin and *p* < 0.001 in all other cases) (Figure 3A). The intracellular concentration of MDA showed a significant increase following incubation with the higher dose of LTA (*p* = 0.006) and flagellin (*p* < 0.001) compared to the control group (Figure 3B).

### 3.2. Intestinal Explant Cultures

**Figure 4:**
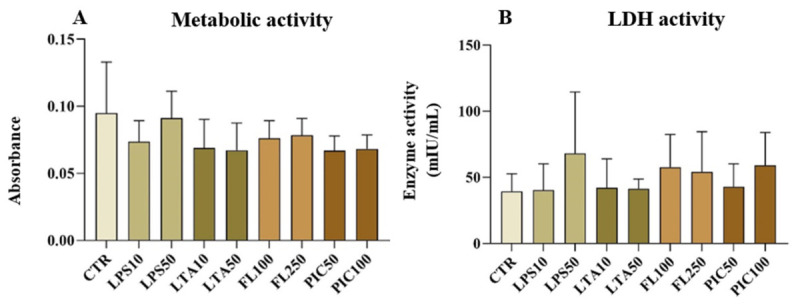
(**A**) Metabolic activity and (**B**) extracellular lactate dehydrogenase (LDH) activity of porcine small intestinal explant cultures, assessed with the CCK-8 test (**A**) and a kinetic photometric assay (**B**). Absorbance (**A**) and enzyme activity (mIU/mL, (**B)**) values are indicated on the y-axes as mean + standard error of the mean. LPS10 and LPS50 refer to 10 and 50 µg/mL lipopolysaccharide (LPS) from *Escherichia coli* (O55:B5), LTA10 and LTA50 indicate 10 and 50 µg/mL lipoteichoic acid (LTA) from *Staphylococcus aureus*, FL100 and FL250 denote 100 or 250 ng/mL flagellin from *Salmonella* Typhimurium, while PIC50 and PIC100 indicate 50 and 100 µg/mL polyinosinic–polycytidylic acid (poly I:C) exposures. *n* = 6 per group in all cases.

The metabolic activity and extracellular LDH activity of small intestinal explants were not affected by any of the applied treatments when compared to those of controls (Figure 4A,B).

**Figure 5:**
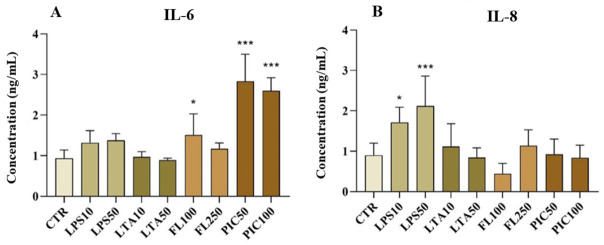
The extracellular concentrations of (**A**) interleukin (IL)-6 and (**B**) IL-8 of porcine small intestinal explant cultures, assessed with Luminex xMAP technology. Concentrations (ng/mL) are indicated on the y-axes as mean + standard error of the mean. LPS10 and LPS50 refer to 10 and 50 µg/mL lipopolysaccharide (LPS) from *Escherichia coli* (O55:B5), LTA10 and LTA50 indicate 10 and 50 µg/mL lipoteichoic acid (LTA) from *Staphylococcus aureus*, FL100 and FL250 denote 100 or 250 ng/mL flagellin from *Salmonella* Typhimurium, while PIC50 and PIC100 indicate 50 and 100 µg/mL polyinosinic–polycytidylic acid (poly I:C) exposures. *n* = 6 per group in all cases. * *p* < 0.05 and *** *p* < 0.001 compared to the controls.

Among the investigated inflammatory cytokines, only IL-6 and IL-8 concentrations were measurable in the explant culture medium, whereas the levels of IL-4, TNF-α, GM-CSF and IFN-γ were below the detection limits of the applied Luminex xMAP assay. Tissue cultures released significantly higher amounts of IL-6 into the supernatant following exposure to 100 ng/mL flagellin (*p* = 0.033), as well as after treatment with both concentrations of poly I:C (*p* < 0.001 in both cases) (Figure 5A). The IL-8 concentration of the culture medium was significantly increased after incubation with both LPS doses (*p* = 0.011 and *p* < 0.001) (Figure 5B).

## 4. Discussion

The present study demonstrates that certain PAMPs can effectively provoke inflammatory and redox responses in porcine in vitro models. However, the extent and characteristics of the triggered responses substantially differ between hepatic co-cultures and small intestinal explant cultures. By integrating the findings obtained in these two models, important insights can be gained into tissue-specific immune reactivity and the suitability of these systems for mimicking inflammatory processes in swine, while also comparing the mechanisms with those of other species.

In the porcine hepatic co-culture, PAMP stimulation elicited a robust inflammatory response, largely attributable to the presence of the macrophage-enriched NP cell fraction in co-cultures. Based on the results of the present study and those of our previous trials with porcine [8] and chicken-derived [2,5] hepatic co-cultures, the applied 6:1 hepatocyte–NP cell ratio, reflecting a moderately inflamed hepatic microenvironment. Therefore, it can be considered an appropriate and physiologically relevant model for subsequent investigations.

Regarding cellular viability, most PAMP treatments did not compromise metabolic activity, with the notable exception of high-dose poly I:C, which evoked significant metabolic suppression. Nevertheless, elevated extracellular LDH activity was observed following exposure to LPS, LTA and poly I:C, indicating increased membrane permeability. In the case of poly I:C, this effect was more pronounced and was associated with overt cytotoxicity, whereas for LPS and LTA, the preserved metabolic activity and concurrent cytokine production suggest sublethal membrane leakage without loss of overall metabolic viability. These findings indicate that LPS and LTA can be reliably applied to induce inflammation in this model without confounding cytotoxic effects.

Among the tested PAMPs, LPS and LTA emerged as the most potent inducers of pro-inflammatory cytokine release, significantly elevating extracellular IL-4, IL-6, IL-8 and TNF-α levels. The pronounced responsiveness to LPS is consistent with its well-established role as a TLR4 agonist and as a classical inducer of inflammation across multiple cell types. It was previously demonstrated by our research group that LPS stimulation increased IL-6 and IL-8 production of porcine hepatocyte mono-cultures, as well as that of enterohepatic co-cultures [21]. Moreover, several studies have reported the pro-inflammatory effects of LPS in the porcine jejunum-derived IPEC-J2 cell line [21,22,23]. In contrast, LPS failed to elicit a cytokine response in our previous experiment conducted in chicken hepatic co-cultures [2], which is likely attributable to avian-specific differences in the TLR4 signaling pathway [19,20]. In avian species, TLRs specialized for the recognition of extracellular pathogens exclusively activate the myeloid differentiation primary response protein 88 (MyD88)-dependent signaling pathway, which is required for the activation of nuclear factor-κB (NF-κB). However, in humans, LPS induces pro-inflammatory cytokine production and delayed NF-κB activation via a MyD88-independent pathway, which is absent in birds. This hypothesis is supported by the observation that infections with *Salmonella* Enteritidis, *Salmonella* Gallinarum and *Pasteurella multocida* do not trigger interferon production in chickens [24]. Furthermore, the lack of certain orthologs of mammalian TLR4 signaling genes in chickens may also contribute to the absence of an LPS-induced cytokine response [19,20].

The markedly higher extracellular IL-8 concentrations compared with the other cytokines should be interpreted considering the biological role and production kinetics of this mediator. IL-8 is a strongly inducible chemokine responsible for neutrophil recruitment and activation; its release can be particularly pronounced in hepatocyte–macrophage/Kupffer cell co-culture systems. Previous porcine studies demonstrated that LPS significantly enhanced IL-8 production in primary hepatocyte cultures, enterohepatic co-cultures and hepatocyte–Kupffer cell co-cultures, with the presence of Kupffer cells being critical for the development of a strong pro-inflammatory cytokine response [8,25]. Therefore, the high IL-8 concentrations observed after LPS and LTA exposure in the present hepatic co-culture model most likely reflect the strong chemokine-producing capacity of porcine hepatocyte–NP cell cultures.

Another observation was the significant elevation of extracellular IL-4 following LPS and, to a lesser extent, LTA exposure. IL-4 is classically associated with Th-type responses and is not a characteristic product of direct TLR4 or TLR2 signaling in hepatocytes or Kupffer cells [26,27]. The increased IL-4 release observed after LPS and LTA exposure may be more likely related to indirect paracrine interactions within the hepatocyte–NP cell co-culture, including possibly present minor NP cell fractions like natural killer T cells or basophils, rather than to the direct activation of hepatocytes or macrophages alone [28,29]. However, since no experiment-specific immunophenotyping was performed in the present study, the precise cellular origin of IL-4 cannot be determined. Therefore, further studies using additional cell-type-specific characterization would be required to clarify the mechanism underlying IL-4 production in this model.

In contrast to the endotoxins tested, flagellin failed to elicit a measurable cytokine response, suggesting a limited activation of the corresponding signaling pathways in the porcine hepatic model. Poly I:C induced a selective increase in IL-6, but concurrently reduced GM-CSF and IFN-γ levels. The marked decrease in extracellular IFN-γ concentrations may appear unexpected, given that viral nucleic acid analogs are generally reported to induce a pronounced interferon response [30], and similar effects were observed in our previous experiments using chicken-derived hepatic cell models [31,32,33]. The observed poly I:C-triggered diminished GM-CSF and IFN-γ release can be associated with its detected cytotoxic effects, as supported by markedly elevated LDH activity. These observations highlight that, while poly I:C is commonly used to mimic viral infection, its applicability in this porcine hepatic model is limited due to its detrimental impact on cell viability and innate immune function.

The data gained on redox homeostasis further support the close interplay between inflammatory and oxidative stress responses [34]. All tested PAMPs increased H_2_O_2_ production, indicating enhanced ROS generation, which was also described in chicken-derived hepatic cell culture models by our research group [31,32,35,36]. However, lipid peroxidation, as reflected by MDA levels, was only stimulated by higher-dose LTA and flagellin treatments. This dissociation between ROS production and lipid damage, also observed in our previous trials [31,32,36], suggests an effective activation of cellular antioxidant defense mechanisms, likely mediated by the nuclear factor erythroid 2-related factor 2 (Nrf2) pathway, mitigating oxidative injury despite increased oxidative burden [34,37]. Thus, the limited increase in MDA despite enhanced H_2_O_2_ generation may indicate that enzymatic and glutathione-dependent antioxidant systems were sufficient to counteract lipid peroxidation in most treatment groups.

In contrast to the hepatic model, porcine intestinal explants displayed markedly increased resistance to PAMP-induced stress. Neither metabolic activity nor membrane integrity was significantly affected by any of the applied treatments, indicating the absence of cytotoxic effects. This enhanced resilience was accompanied by a substantially attenuated inflammatory response compared to the hepatic cell cultures, as evidenced by limited pro-inflammatory cytokine release. Among the cytokines measured, only IL-6 and IL-8 were detectable in the culture medium, even with the high sensitivity of the Luminex xMAP technology, while IL-4, TNF-α and IFN-γ levels were below the quantification limits of the assay in both control and PAMP-exposed groups. Nevertheless, distinct patterns of the cytokine profile were observed: LPS selectively increased extracellular IL-8 concentrations, while poly I:C and the lower dose of flagellin elevated IL-6 levels, the latter to a lesser extent. These findings suggest that intestinal explants retain the capacity to mount pathogen-specific responses, albeit in a less pronounced manner than hepatic cells. The reduced responsiveness of explant cultures can be associated with the biological adaptation of gut tissue to microbial exposures. However, it might also be related to the shorter incubation time resulting from the limited maintainability of mucosal explants [6], which should be considered a limitation of the model.

The comparison between hepatic and intestinal models highlights clear tissue-specific differences in PAMP sensitivity. The liver model exhibited heightened inflammatory responsiveness but also increased susceptibility to cytotoxic effects, particularly in the case of poly I:C. In contrast, intestinal explants demonstrated greater robustness and a more restrained cytokine output. These differences likely reflect the distinct physiological roles of the two tissues. The liver acts as a central immunological filter with rapid and strong inflammatory signaling, whereas the intestinal mucosa plays a key role in balancing immune activation with tolerance to luminal antigens [38]. The relatively moderate cytokine response of intestinal explants should also be interpreted in light of the inherent limitations of ex vivo tissue culture systems. Although metabolic activity and extracellular LDH activity were not significantly altered in the present study, suggesting preserved overall viability, subtle functional changes during incubation cannot be fully excluded. Previous studies have shown that intestinal explants are highly dependent on adequate oxygenation and nutrient supply, and that prolonged in vitro incubation may compromise tissue integrity and immunological responsiveness, partly due to hypoxia and post-mortem tissue degradation [6,39]. Furthermore, under static culture conditions, mass transport in multilayered or 3D mucosal constructs may be diffusion-limited, which can generate spatial gradients of soluble stimuli and secreted factors; consequently, PAMP exposure of deeper mucosal layers and the resulting cytokine release may be lower than in more directly accessible monolayer culture systems [40,41]. The small size of the explants used in the present study was intended to minimize these limitations [6]. Nevertheless, restricted penetration of the stimulants and partial functional decline of the tissue during incubation may have contributed to the lower responsiveness of the intestinal model.

Comparative observations with chicken-derived primary models established and investigated in previous works of our research group further emphasize species-specific differences in PAMP responsiveness [2,6]. Both porcine and avian intestinal explants showed relative resistance to PAMP-related cytotoxic effects compared to the liver cells. The extracellular LDH activity of chicken-derived small intestinal explants remained unchanged following all PAMP exposures, while their metabolic activity decreased only in response to the higher dose of poly I:C treatment [6]. In contrast, the catabolic metabolism of chicken hepatocytes was suppressed by poly I:C, whereas LDH leakage was elevated by LTA, indicating membrane damage [2]. However, the cytokine response patterns of porcine and chicken explants differed considerably. Notably, chicken models responded intensely to LTA, flagellin and poly I:C, while only LPS and poly I:C induced measurable changes in porcine explants. These discrepancies may be attributed to the aforementioned interspecies variation in TLR expression and downstream signaling pathways [19,20].

## 5. Conclusions

In conclusion, the applied porcine hepatocyte–NP cell co-culture represents a sensitive and reliable in vitro model for studying pathogen-associated inflammatory response in swine. Among the PAMPs tested, the endotoxins LPS and LTA were found to be optimal, non-cytotoxic inducers of robust pro-inflammatory cytokine production, providing a proper, reproducible inflammatory model of the porcine liver. In contrast, porcine intestinal explants are considered a more resilient but less responsive system suitable for investigating moderate, gut-specific immune reactions. The observed differences between hepatic and intestinal models, as well as between species, underscore the importance of model selection in experimental design. Collectively, these findings contribute to a better understanding of tissue-specific innate immune responses and support the complementary use of multiple in vitro systems for the investigation of inflammation.

## Data Availability

The datasets generated and/or analyzed during the current study are available from the corresponding author upon reasonable request.

## Abbreviations

The following abbreviations are used in this manuscript:

ANOVA	Analysis of variance
BSA	Bovine serum albumin
CCK	Cell Counting Kit
DMEM	Dulbecco’s Modified Eagle’s Medium
EDTA	Ethylenediamine–tetraacetic acid
FBS	Fetal bovine serum
GM-CSF	Granulocyte–monocyte colony-stimulating factor
HBSS	Hanks’ Balanced Salt Solution
HEPES	4-(2-hydroxyethyl)-1-piperazineethanesulfonic acid
IFN	Interferon
IL	Interleukin
IU	International unit
LDH	Lactate dehydrogenase
LPS	Lipopolysaccharide
LTA	Lipoteichoic acid
MDA	Malondialdehyde
M-PER	Mammalian Protein Extraction Reagent
NAD^+^	Nicotinamide adenine dinucleotide
NF-κB	Nuclear factor-κB
NP	Non-parenchymal
Nrf2	Nuclear factor erythroid 2-related factor 2
PAMP	Pathogen-associated molecular pattern
PBS	Phosphate-buffered saline
Poly I:C	Polyinosinic–polycytidylic acid
ROS	Reactive oxygen species
TLR	Toll-like receptor
TNF	Tumor necrosis factor

## References

[1] VanderWaal K., Deen J. (2018). Global Trends in Infectious Diseases of Swine. Proc. Natl. Acad. Sci. USA.

[2] Sebők C., Tráj P., Vörösházi J., Mackei M., Papp M., Gálfi P., Neogrády Z., Mátis G. (2021). Two Sides to Every Question: Attempts to Activate Chicken Innate Immunity in 2D and 3D Hepatic Cell Cultures. Cells.

[3] Marks H., Grześkowiak Ł., Martinez-Vallespin B., Dietz H., Zentek J. (2022). Porcine and Chicken Intestinal Epithelial Cell Models for Screening Phytogenic Feed Additives—Chances and Limitations in Use as Alternatives to Feeding Trials. Microorganisms.

[4] Kallapura G.K., Hernandez-Velasco X., Piekarski A., Lassiter K., Pumford N.R., Tellez G., Bottje W.G., Hargis B.M., Faulkner O.B. (2015). Development of an Ex Vivo Ileal Explant Culture Method for Amplified Production and Differential Measurement of Nitrite. Int. J. Poult. Sci..

[5] Mackei M., Molnár A., Nagy S., Pál L., Kővágó C., Gálfi P., Dublecz K., Husvéth F., Neogrády Z., Mátis G. (2020). Effects of Acute Heat Stress on a Newly Established Chicken Hepatocyte—Nonparenchymal Cell Co-Culture Model. Animals.

[6] Mátis G., Sebők C., Horváth D.G., Márton R.A., Mackei M., Vörösházi J., Kemény Á., Neogrády Z., Varga I., Tráj P. (2025). Miniature Chicken Ileal Explant Culture to Investigate the Inflammatory Response Induced by Pathogen-Associated Molecular Patterns. Front. Vet. Sci..

[7] Hoebe K.H., Monshouwer M., Witkamp R.F., Fink-Gremmels J., van Miert A.S. (2000). Cocultures of Porcine Hepatocytes and Kupffer Cells as an Improved in Vitro Model for the Study of Hepatotoxic Compounds. Vet. Q..

[8] Mátis G., Kulcsár A., Petrilla J., Talapka P., Neogrády Z. (2017). Porcine Hepatocyte–Kupffer Cell Co-Culture as an in Vitro Model for Testing the Efficacy of Anti-Inflammatory Substances. J. Anim. Physiol. Anim. Nutr..

[9] Wu L., Ferracci G., Wang Y., Fan T.F., Cho N.-J., Chow P.K.H. (2019). Porcine Hepatocytes Culture on Biofunctionalized 3D Inverted Colloidal Crystal Scaffolds as an in Vitro Model for Predicting Drug Hepatotoxicity. RSC Adv..

[10] Talbot N.C., Caperna T.J., Lebow L.T., Moscioni D., Pursel V.G., Rexroad C.E. (1996). Ultrastructure, Enzymatic, and Transport Properties of the PICM-19 Bipotent Liver Cell Line. Exp. Cell Res..

[11] Schierack P., Nordhoff M., Pollmann M., Weyrauch K.D., Amasheh S., Lodemann U., Jores J., Tachu B., Kleta S., Blikslager A. (2006). Characterization of a Porcine Intestinal Epithelial Cell Line for in Vitro Studies of Microbial Pathogenesis in Swine. Histochem. Cell Biol..

[12] Cortez B.R.d.S., Guedes R.M.C. (2023). A Review on the Evolution of Methods for Intestinal in Vitro Organ Culture and Its Application in Veterinary Science. Vet. World.

[13] Ahmed A.U. (2011). An Overview of Inflammation: Mechanism and Consequences. Front. Biol..

[14] Mifsud E.J., Tan A.C.-L., Jackson D.C. (2014). TLR Agonists as Modulators of the Innate Immune Response and Their Potential as Agents Against Infectious Disease. Front. Immunol..

[15] Morath S., Stadelmaier A., Geyer A., Schmidt R.R., Hartung T. (2002). Synthetic Lipoteichoic Acid from Staphylococcus Aureus Is a Potent Stimulus of Cytokine Release. J. Exp. Med..

[16] Kang J.Y., Lee J.-O. (2011). Structural Biology of the Toll-Like Receptor Family. Annu. Rev. Biochem..

[17] Akira S., Uematsu S., Takeuchi O. (2006). Pathogen Recognition and Innate Immunity. Cell.

[18] Kaschubek T., Mayer E., Rzesnik S., Grenier B., Bachinger D., Schieder C., König J., Teichmann K. (2018). Effects of Phytogenic Feed Additives on Cellular Oxidative Stress and Inflammatory Reactions in Intestinal Porcine Epithelial Cells. J. Anim. Sci..

[19] Kannaki T.R., Reddy M.R., Shanmugam M., Verma P.C., Sharma R.P. (2010). Chicken Toll-like Receptors and Their Role in Immunity. World’s Poult. Sci. J..

[20] Kim J.-K., Lee S.-M., Suk K., Lee W.-H. (2011). A Novel Pathway Responsible for Lipopolysaccharide-Induced Translational Regulation of TNF-α and IL-6 Expression Involves Protein Kinase C and Fascin. J. Immunol..

[21] Farkas O., Mátis G., Pászti-Gere E., Palócz O., Kulcsár A., Petrilla J., Csikó G., Neogrády Z., Gálfi P. (2014). Effects of Lactobacillus Plantarum 2142 and Sodium N-Butyrate in Lipopolysaccharide-Triggered Inflammation: Comparison of a Porcine Intestinal Epithelial Cell Line and Primary Hepatocyte Monocultures with a Porcine Enterohepatic Co-Culture System. J. Anim. Sci..

[22] Zhao X., Dong B., Friesen M., Liu S., Zhu C., Yang C. (2021). Capsaicin Attenuates Lipopolysaccharide-Induced Inflammation and Barrier Dysfunction in Intestinal Porcine Epithelial Cell Line-J2. Front. Physiol..

[23] Cai L., Wei Z., Zhao X., Li Y., Li X., Jiang X. (2022). Gallic Acid Mitigates LPS-Induced Inflammatory Response via Suppressing NF-κB Signalling Pathway in IPEC-J2 Cells. J. Anim. Physiol. Anim. Nutr..

[24] Keestra A.M., van Putten J.P.M. (2008). Unique Properties of the Chicken TLR4/MD-2 Complex: Selective Lipopolysaccharide Activation of the MyD88-Dependent Pathway. J. Immunol..

[25] Paszti-Gere E., Matis G., Farkas O., Kulcsar A., Palocz O., Csiko G., Neogrady Z., Galfi P. (2014). The Effects of Intestinal LPS Exposure on Inflammatory Responses in a Porcine Enterohepatic Co-Culture System. Inflammation.

[26] Nakamoto N., Kanai T. (2014). Role of Toll-Like Receptors in Immune Activation and Tolerance in the Liver. Front. Immunol..

[27] Keegan A.D., Leonard W.J., Zhu J. (2021). Recent Advances in Understanding the Role of IL-4 Signaling. Fac. Rev..

[28] Wang H., Feng D., Park O., Yin S., Gao B. (2013). Invariant NKT Cell Activation Induces Neutrophil Accumulation and Hepatitis: Opposite Regulation by IL-4 and IFN-γ. Hepatology.

[29] Bandyopadhyay K., Marrero I., Kumar V. (2016). NKT Cell Subsets as Key Participants in Liver Physiology and Pathology. Cell. Mol. Immunol..

[30] Matsumoto M., Seya T. (2008). TLR3: Interferon Induction by Double-Stranded RNA Including Poly(I:C). Adv. Drug Deliv. Rev..

[31] Márton R.A., Sebők C., Mackei M., Tráj P., Vörösházi J., Kemény Á., Neogrády Z., Mátis G. (2024). Pap12-6: A Host Defense Peptide with Potent Immunomodulatory Activity in a Chicken Hepatic Cell Culture. PLoS ONE.

[32] Márton R.A., Sebők C., Mackei M., Tráj P., Vörösházi J., Kemény Á., Neogrády Z., Mátis G. (2024). Cecropin A: Investigation of a Host Defense Peptide with Multifaceted Immunomodulatory Activity in a Chicken Hepatic Cell Culture. Front. Vet. Sci..

[33] Tráj P., Herrmann E.M., Sebők C., Vörösházi J., Mackei M., Gálfi P., Kemény Á., Neogrády Z., Mátis G. (2022). Protective Effects of Chicoric Acid on Polyinosinic-Polycytidylic Acid Exposed Chicken Hepatic Cell Culture Mimicking Viral Damage and Inflammation. Vet. Immunol. Immunopathol..

[34] Rushworth S.A., Chen X.-L., Mackman N., Ogborne R.M., O’Connell M.A. (2005). Lipopolysaccharide-Induced Heme Oxygenase-1 Expression in Human Monocytic Cells Is Mediated via Nrf2 and Protein Kinase C. J. Immunol..

[35] Sebők C., Tráj P., Mackei M., Márton R.A., Vörösházi J., Kemény Á., Neogrády Z., Mátis G. (2023). Modulation of the Immune Response by the Host Defense Peptide IDR-1002 in Chicken Hepatic Cell Culture. Sci. Rep..

[36] Sebők C., Walmsley S., Tráj P., Mackei M., Vörösházi J., Petrilla J., Kovács L., Kemény Á., Neogrády Z., Mátis G. (2022). Immunomodulatory Effects of Chicken Cathelicidin-2 on a Primary Hepatic Cell Co-Culture Model. PLoS ONE.

[37] Lee I.-T., Wang S.-W., Lee C.-W., Chang C.-C., Lin C.-C., Luo S.-F., Yang C.-M. (2008). Lipoteichoic Acid Induces HO-1 Expression via the TLR2/MyD88/c-Src/NADPH Oxidase Pathway and Nrf2 in Human Tracheal Smooth Muscle Cells. J. Immunol..

[38] Kubes P., Jenne C. (2018). Immune Responses in the Liver. Annu. Rev. Immunol..

[39] Bahar B., O’Doherty J.V., Vigors S., Sweeney T. (2016). Activation of Inflammatory Immune Gene Cascades by Lipopolysaccharide (LPS) in the Porcine Colonic Tissue Ex-Vivo Model. Clin. Exp. Immunol..

[40] Dufva M. (2023). A Quantitative Meta-Analysis Comparing Cell Models in Perfused Organ on a Chip with Static Cell Cultures. Sci. Rep..

[41] Makkar H., Sriram G. (2025). Advances in Modeling Periodontal Host–Microbe Interactions: Insights from Organotypic and Organ-on-Chip Systems. Lab Chip.

